# Expression of the T regulatory cell transcription factor FoxP3 in peri-implantation phase endometrium in infertile women with endometriosis

**DOI:** 10.1186/1477-7827-10-34

**Published:** 2012-04-27

**Authors:** Shufang Chen, Jian Zhang, Changxiao Huang, Wen Lu, Yan Liang, Xiaoping Wan

**Affiliations:** 1 Department of Gynecology, International Peace Maternity and Child Health Hospital, School of Medicine, Shanghai Jiaotong University, Shanghai, China

**Keywords:** Endometriosis, FoxP3, Infertility, T regulatory cells

## Abstract

**Background:**

Endometriosis (EM) is highly associated with infertility. The precise mechanism underlying EM-associated infertility remains controversial. This study aimed to investigate the pathogenesis of infertility in women with EM by comparing FoxP3+ T regulatory cells (Tregs) expression in the eutopic endometrium of infertile women with EM and endometrium from healthy fertile women.

**Methods:**

As a marker of Tregs**,** FoxP3 expression was analyzed in eutopic endometrium during the peri-implantation phase in infertile women with mild EM (n = 7), advanced EM (n = 20), and normally fertile women without EM (n = 20). *FoxP3* mRNA expression was analyzed by quantitative real-time RT-PCR. FoxP3 protein expression was assessed by immunohistochemistry.

**Results:**

*FoxP3* mRNA expression in all infertile patients with EM was significantly higher than the control group (*P* < 0.05) by non-parametric Mann–Whitney *U*-test. Further analysis based on the extent of EM revealed that *FoxP3* mRNA expression in infertile patients with advanced EM was significantly higher than the mild EM group and the control group (*P* < 0.05). Immunohistochemistry analysis showed predominant positive staining for FoxP3 protein in the endometrial stroma. In addition, the number of FoxP3+ cells in the eutopic endometrium of infertile women with advanced EM was marginally higher than the mild EM group and the control group, although the differences were not statistically significant (*P* > 0.05) by two-tailed *t*-tests.

**Conclusions:**

These findings suggest that FoxP3+ Tregs in the peri-implantation endometrium might participate in the pathogenesis of advanced EM. However, they are not directly involved in the pathogenesis of advanced EM associated with infertility. The differential expression of FoxP3 in infertile women with mild EM and advanced EM implicates that notable differences in the uterine immune status are likely involved in the pathogenesis of mild EM associated with infertility in the peri-implantation endometrium.

## Background

Endometriosis (EM) is a common and benign gynecological disorder that is highly associated with infertility. It affects approximately 10% to 15% of women of reproductive age and 25% to 50% of women with infertility. Moreover, 30% to 50% of women with EM are infertile [1]. Although the mechanisms underlying EM-associated infertility include abnormal folliculogenesis, elevated oxidative stress, altered immune function and hormonal milieu in the follicular and peritoneal environments, and reduced endometrial receptivity, the precise mechanism of pathogenesis remains controversial. The combination of these factors leads to poor oocyte quality and impaired fertilization and implantation [2,3]. Recent studies have demonstrated that endometrial molecular defects during the implantation window might be a cause of EM-associated infertility. Increasing evidence suggests that EM patients have an impaired endometrium and/or an abnormal endometrial environment which make them functionally unfavorable for implantation and pregnancy progression [2,4].

CD4^+^CD25^+^FoxP3^+^T regulatory cells (T_regs_) are a specialized subpopulation of T cells that control and suppress a range of immune responses, including T-cell proliferation and activation, macrophage, B cell, DC and NK cell function, mast cell degranulation, cell proliferation, and cytokine release. Forkhead box protein 3 (FoxP3) is a member of the forkhead-box/winged-helix transcription factor family. It is a unique marker of T_regs_. FoxP3 has been reported to be an essential controlling gene for the development and function of naturally occurring T_reg_ populations.

Accumulating evidence from both experimental and clinical studies indicates that a balance between regulation and deletion of responder T cells is an effective strategy to control immune responsiveness after organ or cell transplantation [5]. Furthermore, FoxP3^+^ T_regs_ are critical for the maintenance of maternal immune tolerance as well as the prevention of autoimmunity and transplantation rejection. Recent studies have demonstrated links between impaired function or diminished T_reg_ cell populations and complications during pregnancy due to defective implantation or placental insufficiency [6]. In miscarriage, reduced responsiveness to pregnancy associated expansion of T_reg_ cell populations, due to numerically fewer T_regs_ as well as T_reg_ functional deficiency, may underpin reduced immunosuppressive capability [7]. Compared to women with induced abortion, patients experiencing spontaneous abortion exhibit fewer decidual and peripheral blood CD4^+^CD25^high^ T cells recovery. Women experiencing repeated miscarriage have been shown to have a reduced number of T_regs_ within the peripheral blood CD4^+^ pool and reduced suppressive capacity. Primary unexplained infertility is also associated with reduced expression of FoxP3 mRNA in endometrial tissue during mid-secretory phase of the menstrual cycle, suggesting that impaired differentiation and/or recruitment of uterine T_regs_, even prior to conception, might affect patients’ ability to establish pregnancy [8].

A recent study demonstrated that eutopic endometrial FoxP3 was up-regulated in women with EM, suggesting that FoxP3 plays a pathogenic role in the formation of EM [9]. However, very little is known about the role of FoxP3^+^ T_regs_ in the pathophysiologic mechanism of EM-associated infertility and the changes of FoxP3^+^ T_reg_ population in different EM stages. In this study, FoxP3 expression in the endometrium during the peri-implantation phase was investigated by comparing infertile women with different stages of EM to normal fertile women. The purpose is to elucidate the pathogenesis of infertility in EM.

## Methods

### Patients and samples

All subjects were patients admitted in the Gynecology Department of the International Peace Maternity and Child Health Hospital, Shanghai Jiaotong University (China) between April 2009 and July 2010. There were 27 primary infertile women with regular menstrual cycles in the study group. All women had visual or biopsy-proven EM. They had undergone endometrial curettage and laparoscopic excision of endometriotic ovarian cysts or endometriotic peritoneal lesions between days 19 and 23 of the cycle. Procedures were performed based on endometrial histological dating [10] and the first day of their last menstrual period (LMP). Infertile women with EM exhibited a minimum 1 year of infertility with a current desire for conception, no chromosomal anomalies in either parent, no uterine structural abnormalities, no thrombophilic disorders, and no contribution of male factor infertility. The control group consisted of 20 women without pelvic EM, confirmed during laparoscopic surgery for para-ovarian cysts or mesosalpinx cysts. All of them had regular menstrual cycles and successful pregnancies. Endometrial biopsies from the control group were taken between days 19 and 23 of the cycle with dating confirmed by microscopic examination and LMP. All samples were histologically examined by a histopathology specialist. The extent of EM was staged according to the revised American Society for Reproductive Medicine classification (rAFS) system [11].

None of the patients in the study group and control group had received any hormone therapy within 6 months of the procedure, had experienced a miscarriage, or had a history of in vitro fertilization treatment. All women abstained from intercourse or used barrier methods of contraception for the period between their last menses and sample collection. Exclusion criteria included: history of autoimmune diseases, pelvic inflammatory disease, genital tract infection, use of intrauterine contraception for at least 6 months prior to surgery, endometrial hyperplasia or endometrial polyps, and concomitant adenomyosis and uterine fibroids. All women in the control group had only one living child and had no history of spontaneous abortion, ectopic pregnancy, or preterm delivery.

This study was approved by the human ethics committee of the International Peace Maternity and Child Health Hospital. A written informed consent form was obtained from each participant prior to their inclusion.

### Tissue collection

On the day of operation, endometrial tissue was obtained by curettage. Each biopsy was divided into two portions. One was snap-frozen in liquid nitrogen and stored at −80°C until further analysis was performed. The other portion was fixed in 10% neutral buffer formalin for 18 to 24 hours and was embedded in paraffin for further histological dating and immunohistochemical analysis. Plasma progesterone levels were measured to ensure that ovulation had occurred.

### Tissue processing

#### Quantitative real-time PCR (qRT-PCR)

Total RNA was extracted using Trizol method (Invitrogen) from the homogenized tissues, and then quantified on a NanoDrop™ 1000 Spectrophotometer (Thermo scientific Waltham, MA, USA). The OD260nm/280 nm ratio was among 1.9 to 2.0. RNA samples were further assessed by electrophoresis on 1.5% agarose gels, and then visualized under UV light after ethidium bromide staining. RNA samples were stored at −80°C in aliquots until use.

cDNA was synthesized with 1 μg total RNA and 1 μl d(T)18 Oligo using RevertAid First Strand cDNA Synthesis Kit (Fermentas Thermo). Final volume was 20 μl. *FoxP3* transcripts were relatively quantified by real-time RT-PCR with SYBR-Green master mix (ABI) on 7500 Real-time PCR System (Applied Biosystems, CA, USA). Reaction mixtures, in a total volume of 10 μl, contained 5 μl SYBR Green, 0.15 μl primer (Table 1), 1 μl cDNA (10 fold diluted) and 3.85 μl RNase-free water. As a negative control, H_2_O was used instead of cDNA. The PCR was carried out as follows: 95°C for 2 min, 40 cycles of 95°C for 15 s, and 60°C for 1 min. A melting curve was performed to check the specificity of amplification. The relative transcript concentration was calculated by 2-ΔΔCt taking GAPDH as interval standard control. Each sample was analyzed in triplicate.

**Table 1:** Primer sequences

**Primer**	**Sequence**	**Genbank**
GAPDH	5'- ATGGAAATCCCATCACCATCTT -3'	
	3'- CGCCCCACTTGATTTTGG -5'	
*FoxP3*	5'-TGCAGGGCAGCTAGGTACTTG -3'	NM_014009.3
	3'-TCGGAGATCCCCTTTGTCTTATC -5'	

#### Immunohistochemistry

Endometrial tissues were fixed, cut, mounted, deparaffinized, and rehydrate. After blocking with goat serum, the sections were incubated with the primary murine monoclonal anti-human FoxP3 antibody (Abcam, 236A/E7, Hong Kong) at 4°C overnight (1:50 dilution). After incubation with secondary polyperoxidase-anti-mouse IgG antibody (MR-biotech, Shanghai, China), a horseradish peroxidase detection system was applied. Immunoreactivity was detected using the diaminobenzidine tetrahydrochloride chromogen (MR-biotech, Shanghai, China). Sections were counterstained with hematoxylin, dehydrated and cleared. For a negative control, PBS was substituted for the primary antibody in the above protocols. A sample of lymph node tissue, known to contain FoxP3^+^ cells, was used as a positive control.

The sections were viewed using a light microscope (Zeiss MIC00958) under × 400 magnification (×40 objective, ×10 ocular). T_regs_ were characterized by the brown intracellular staining of FoxP3 antibody in the stroma of the endometrium. FoxP3^+^ cell counting was performed on 10 non-overlapping fields for each sample. Each slide was counted by two different observers blinded to the tissue origin. The average counting from each observer was calculated and a mean was calculated.

#### Statistical analysis

Statistical analysis was performed with GraphPad Prism Version 5. Biological parameters were presumed to exist in a normal distribution. Therefore, two-tailed *t*-tests were used to test significance, and the results were reported as means ± SD. As the results for *FoxP3* mRNA expression did not conform to the normal distribution, the differences among groups were also assessed with a non-parametric Mann–Whitney *U*-test, and results were reported as the median and interquartile range. For all tests, *P* < 0.05 was considered statistically significant.

## Results

### Basic clinical characteristics of patients

The staging of EM in the study group followed the rAFS classification. Patients were divided into two subgroups as follows: 7 patients with mild EM (stage I and II), and 20 with advanced EM (stage III and IV). The mean ages of patients in these two groups were 31.0 years (range, 27–40 years) and 29.8 years (range, 26–37 years), respectively. The mean durations of infertility were 2.85 years (range, 1–6 years) and 3.27 years (range, 1–8 years), respectively. The mean age in the control group (normally fertile women without EM) was 30.9 years (range, 25–37 years). No significant differences were noted in age, cycle length (mild EM 29.98 ± 1.32 vs. advanced EM 31.01 ± 1.41 vs. control 30.10 ± 1.20 days) or timing of sampling (mild EM 21.07 ± 1.60 vs. advanced EM 20.97 ± 1.98 vs. control 21.45 ± 1.35 days) among these groups. Furthermore, serum progesterone levels were similar in these groups (mild EM 50.34 ± 2.59 vs. advanced EM 51.67 ± 2.37 vs. control 52.10 ± 3.02 nmol/L). The demographic details of the study group and control group are summarized in Table 2.

**Table 2:** Clinical characteristics of all women in the study

	**Mild-EM (n = 7)**	**Advanced-EM (n = 20)**	**Control (n = 20)**
Age (years)	31.00 ± 4.31	29.81 ± 3.71	30.92 ± 4.48
Parity	0^a^	0^a^	1^b^
Cycle length (days)	29.98 ± 1.32	31.01 ± 1.41	30.10 ± 1.20
Progesterone level (nmol/l)	50.34 ± 2.59	51.67 ± 2.37	52.10 ± 3.02
Timing of sampling (days)	21.07 ± 1.60	20.97 ± 1.98	21.45 ± 1.35
Histroy of infertility (years)	2.85 ± 2.23	3.27 ± 2.60	0

Values represent mean ± SD.

Mild-EM, infertile women with mild endometriosis.

Advanced-EM, infertile women with advanced endometriosis.

^a^ Primary infertility.

^b^ Had only one child.

### Quantitative real-time PCR analysis of FoxP3 mRNA expression

**Figure 1:**
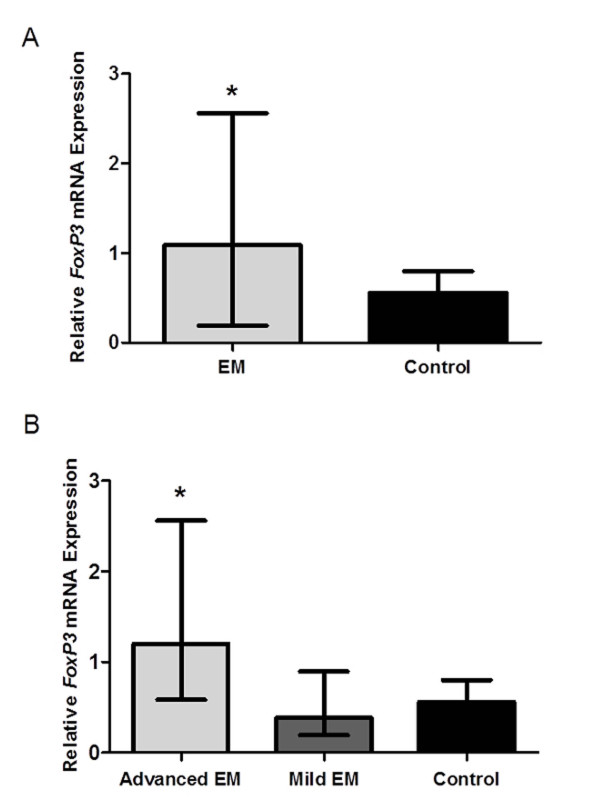
**Quantitative real-time RT-PCR analysis of *FoxP3* mRNA expression in peri-implantation phase eutopic endometrium.** (**a**) Relative expression of *FoxP3* mRNA determined by RT-PCR in peri-implantation phase eutopic endometrium (cycle days 19–23) from infertile women with endometriosis (n = 27) and normal fertile women without endometriosis (n = 20). *Significant difference compared endometriosis group with control group by the Mann–Whitney *U*-test (*P* < 0.05). (**b**) Relative expression of *FoxP3* mRNA determined by RT-PCR in peri-implantation phase eutopic endometrium (cycle days 19–23) from infertile women with mild endometriosis (n = 7), advanced endometriosis (n = 20) and normal fertile women without endometriosis (n = 20). *Significant difference compared advanced endometriosis group with mild endometriosis group and control group by the Mann–Whitney *U*-test (*P* < 0.05). All values were expressed as median (range). (EM, infertile women with endometriosis; Mild EM, infertile women with mild endometriosis; Advanced EM, infertile women with advanced endometriosis).

*FoxP3* mRNA expression in the study group (median, 1.09; interquartile range, 0.58-1.43) was significantly higher than the control group (median, 0.56; interquartile range, 0.21-0.72; *P* < 0.05). Further analysis based on the extent of EM revealed that *FoxP3* mRNA expression in infertile patients with advanced EM (median, 1.20; interquartile range, 0.86-1.95) was significantly higher than the mild EM group (median, 0.38; interquartile range, 0.21-0.47) and the control group (*P* < 0.05). The median level of *FoxP3* mRNA in infertile patients with mild EM was marginally lower than in the control group (0.38 vs. 0.56), although no statistically significant difference was detected between these two groups (*P* > 0.05) (Figure 1).

### Immunohistochemical staining of FoxP3 protein in human endometrium

**Figure 2:**
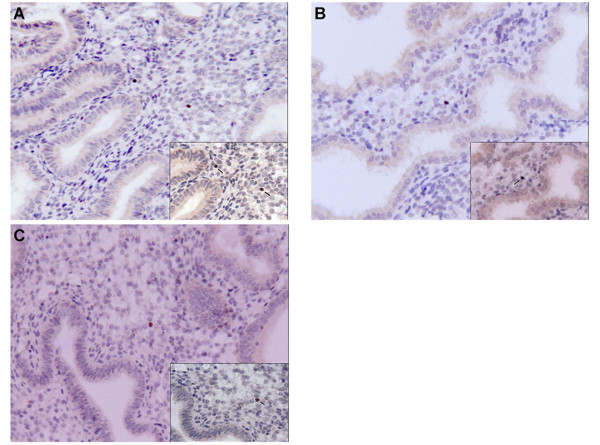
**Immunohistochemical staining of FoxP3 protein in peri-implantation phase eutopic endometrium. **Immunohistochemical staining of FoxP3 protein expression in eutopic endometrium during the peri-implantation phase. Positive immunolabeling for FoxP3 (brown diaminobenzidine chromogen coloration) occurs in the endometrial stroma (arrows). (**a**) FoxP3 protein expression in the endometrium of infertile patients with advanced endometriosis. (**b**) FoxP3 protein expression in the endometrium of infertile patients with mild endometriosis. (**c**) FoxP3 expression in the endometrium of the control group. No significant difference was observed among these groups. (all pictures, original magnification × 200; inset, original magnification × 400).

Immunohistochemical staining showed that FoxP3 protein was predominantly expressed in the endometrial stroma (Figure 2). Enumeration of FoxP3^+^ T_regs_ was expressed as the mean number (± SD) of FoxP3^+^ cells per square millimeter of endometrium. The number of FoxP3^+^ cells in eutopic endometrium of infertile women with advanced EM (0.79 ± 0.52) was marginally higher than the mild EM group (0.50 ± 0.29) and the control group (0.51 ± 0.30), although there were no statistically significant differences among these groups (*P* > 0.05) (Table 3).

**Table 3:** FoxP3 levels in the endometrium

**Groups**	**No.**	FoxP3 (mean ± SD)^*^
Mild-EM	7	0.50 ± 0.29
Advanced-EM	20	0.79 ± 0.52
Control	20	0.51 ± 0.30

Notes: * mean number of FoxP3 + cells per square millimetre of endometrium.

P > 0.05 Mild-EM versus Advanced-EM versus Control.

Mild-EM, infertile women with mild endometriosis.

Advanced-EM, infertile women with advanced endometriosis.

## Discussion

The mechanisms by which EM impairs fertility remain poorly understood. Accumulating evidence indicates that the eutopic endometrium of women with EM differs from that of women without EM [12], which may contribute to failure of implantation. A meta-analysis of in-vitro fertilization and embryo transfer (IVF-ET) trials showed that women with EM have similar ovulation and embryo formation rates compared to patients scheduled for IVF treatment without EM (e.g. blocked fallopian tubes). However, the implantation rates in women with EM are 50% lower than those achieved in patients being treated for other causes of infertility [13]. These results indicate a receptivity defect within the eutopic endometrium in women with EM that affects fertility regardless of other causes of infertility in EM (e.g. adhesions). Gene array studies have established aberrant gene expression in the endometrium of women with EM compared to those without EM during the implantation window [14]. Furthermore, studies indicate that an abnormal inflammatory environment is present, not only in pelvic endometriotic lesions, but also in the eutopic endometrium of patients with EM. Therefore, the decrease in fertility experienced by these women might be caused by inflammatory processes, which in turn, affecting ovulation and implantation.

Successful embryo implantation is a dynamic process, requiring dialog between the blastocyst and a receptive endometrium [15]. Although implantation is primarily regulated by the steroid hormones, a host of local immune cells, cytokines, growth factors and adhesion molecules have been identified that mediate the apposition, adhesion and invasion of the blastocyst [16,17]. Maintenance of an optimal pro- and anti-inflammatory state at the feto-maternal interface is necessary for successful implantation. The leukocyte population in the endometrial environment at the time of implantation includes uterine natural killer (uNK), macrophages, T cells and B cells [18-20]. Dysregulation in the production of these factors may lead to aberrant implantation. As one of these factors, T_regs_ play a crucial role in regulation and suppression of local immune response during implantation phase.

Recently, studies in reproductive immunology show that T_regs_ play an important role in maternal tolerance of the conceptus. Their suppressive actions are exerted even prior to embryo implantation. T_regs_ are enriched at the fetal-maternal interface, showing a suppressive phenotype. Inadequate numbers of T_regs_ or their functional deficiency might be linked with miscarriage, pre-eclampsia, infertility and the failure of embryo implantation. Several studies have reported an association between T_regs_ and implantation failure or recurrent spontaneous miscarriage in humans. Women experiencing repeated miscarriage were shown to have a reduced frequency of T_regs_ within peripheral blood, and reduced suppressive capacity, compared to normal fertile women [7,21]. Primary unexplained infertility has also been associated with reduced expression of *Foxp3* mRNA in endometrial tissue in the mid-secretory phase of the menstrual cycle [8]. These studies suggest that reduction in the size and functional impairment of the T_reg_ population and/or insufficient migration of T_regs_ to decidual tissue at the feto–maternal interface induce implantation failure in embryo implantation or recurrent spontaneous abortion in humans.

In contrast to other leukocytes, T_regs_ play the most crucial roles in controlling, suppressing and modulating a vast variety of immune responses in the development of endometriosis. Endometriosis is an inflammatory condition, associated with highly dysregulated immune response at both uterine and peritoneal levels. Recent evidence suggests that dysregulated immune response in EM is likely to originate within the eutopic endometrium [22]. Berbic et al. found that FoxP3^+^ cells in the eutopic endometrium of women with EM remained highly up-regulated during the secretory phase of the menstrual cycle, while at this time their expression was significantly down-regulated in women without EM [21]. They propose that FoxP3^+^ cells in eutopic endometrium in women with EM decrease the ability of newly recruited immune cell populations to effectively recognize and target endometrial antigens shed during menstruation, allowing their survival and ability to implant in ectopic sites [9]. T_regs_ are likely to be linked to pathogenesis and progression of EM. Basta et al. demonstrated that the disturbance in the immunological equilibrium observed in ectopic endometrium and deciduas would seem to be related to the alteration in the T_reg_ cell population that occurs in these ectopic tissues. Additionally, no differences in the percentage of T_regs_ within the T lymphocyte subpopulation were observed over the course of the menstrual cycle in the ovarian endometriosis. They hypothesized that the absence of T_regs_ fluctuation can be linked to an immune defect arising with the development of endometriosis [23]. In another genetic marker research,André GM et al. first evaluated the association between *FOXP3* polymorphisms in infertile women with and without EM. They suggest that the *FOXP3* polymorphisms can be associated with risk of idiopathic infertility (rs2280883 and rs2232368) and EM (rs3761549) in Brazilian women [24]. In addition, recent studies have implicated T_regs_ in inducing tolerance to tumours. In Prieto’s reviews, the proposed hypothesis predicts that local expansion of T_regs_ might suppress anti-tumour responses and facilitate the progression of EM to ovarian cancer in susceptible women [25].

Very few human studies of FoxP3 expression in the peri-implantation endometrium have been reported. FoxP3 expression in the endometrium of infertile patients with EM compared to healthy fertile women remains to be elucidated. Therefore, this study aimed to study the difference of FoxP3^+^ expression in endometrial tissue during the peri-implantation window between patients with EM-associated infertility and healthy fertile women. Furthermore, FoxP3 expression in the endometrium of infertile patients with different stages of EM and the role of T_regs_ in the etiology of infertility in women with EM were investigated as well. Analysis of *FoxP3* mRNA expression by quantitative real-time RT-PCR revealed that infertile women with EM have higher levels of *FoxP3* mRNA in eutopic endometrium than the control group. Further analysis based on the extent of EM revealed that infertile women with advanced EM have higher levels of *FoxP3* mRNA in eutopic endometrium than women with mild EM and the control group. The results of this study conflict with those reported in 2006 by Jasper et al. [8], which demonstrated an association between unexplained infertility and reduced *FoxP3* mRNA expression in endometrial tissue. However, the results of this study are consistent with Berbic’s report, which demonstrated upregulation of FoxP3 expression in eutopic endometrium in women with EM in the secretory phase [9]. Therefore, it is hypothesized that FoxP3^+^ T_regs_ in the peri-implantation endometrium participate in the pathogenesis of EM while they are not directly involved in the pathogenesis of advanced EM associated infertility.

Additionally, our study showed that the expression of *FoxP3 mRNA* in the infertile women with mild EM was significant lower than patients with advanced EM, but it was similar to the control group. It is suggested by this result that the uterine immune status in peri-implantation endometrium among infertile patients with mild EM is different from that of the advanced EM. This kind of difference might be involved in the failure of embryo implantation and the pathogenesis of infertility in the mild EM. Although there was no statistically significant difference between the mild EM group and the control group, *FoxP3 mRNA* expression in the infertile women with mild EM was slightly lower. It is not clear whether the inadequate numbers of FoxP3^+^ T_regs_ in the peri-implantation endometrium in sub-fertile women with mild EM is related to the pathogenesis of infertility and unsuccessful embryo implantation. This needs to be examined in future studies with large sample size.

T_regs_, which comprise only 5-10% of CD4^+^T cells in human,are few in eutopic endometrium and are periodically regulated by 17-β-estradiol [21]. T_regs_ will increase and exert full suppressive function when they expose to alloantigen, such as embryo, sperm, and inflammation [26,27]. Since our study excluded the effect of alloantigen, the level of FoxP3^+^ cells by semiquantitative immunohistochemical staining was relatively low. Although there was no statistically significant difference, FoxP3^+^ expression in the advanced EM group was higher than the mild EM group and the control group. This result was consistent with the findings in the quantitative real-time PCR analysis. Because it is unethical to investigate human embryo implantation process in vivo, future studies may focus on the changes of T_regs_ during peri-implantation phase in the eutopic endometrium of infertile women with EM in vitro.

## Conclusions

From the above, our findings suggest that FoxP3^+^ T_regs_ in the peri-implantation endometrium might participate in the pathogenesis of advanced EM. However, they are not directly involved in the pathogenesis of advanced EM associated with infertility. The differential expression of FoxP3 in infertile women with mild EM and advanced EM implicates that notable differences in the uterine immune status are likely involved in the pathogenesis of mild EM associated with infertility in the peri-implantation endometrium.

## Abbreviations

EM, Endometriosis; Tregs, CD4+CD25+FoxP3+T regulatory cells; FoxP3, $f(x) = {{a}_0} + \sum\nolimits_{{n = 1}}^{{00}} ( {{a}_n}\cos \frac{{n\pi x}}{L} + {{b}_n}\sin \frac{{n\pi x}}{L})$Forkhead box protein 3; LMP, Last menstrual period; rAFS, Revised American Society for Reproductive Medicine classification system; uNK, Uterine natural killer; IVF-ET, In-vitro fertilization and embryo transfer.

## Competing interests

The authors declare that they have no competing interests.

## Authors’ contributions

SC and JZ contributed to the design of the study, acquisition of data, analysis and interpretation of data, and writing the manuscript. CH, WL and YL were involved in the experimental work of the study. XW was principal project supervisor and was mainly responsible for the intellectual planning of the project. All authors read and approved the final manuscript.
